# Apelin, Elabela/Toddler, and biased agonists as novel therapeutic agents in the cardiovascular system

**DOI:** 10.1016/j.tips.2015.06.002

**Published:** 2015-09

**Authors:** Peiran Yang, Janet J. Maguire, Anthony P. Davenport

**Affiliations:** Experimental Medicine and Immunotherapeutics, Level 6 Addenbrooke's Centre for Clinical Investigation, Box 110 Addenbrooke's Hospital, Cambridge CB2 0QQ, UK

**Keywords:** apelin, Elabela/Toddler, pulmonary arterial hypertension, apelin receptor, APJ, biased agonist

## Abstract

Apelin and its G protein-coupled receptor (GPCR) have emerged as a key signalling pathway in the cardiovascular system. The peptide is a potent inotropic agent and vasodilator. Remarkably, a peptide, Elabela/Toddler, that has little sequence similarity to apelin, has been proposed as a second endogenous apelin receptor ligand and is encoded by a gene from a region of the genome previously classified as ‘non-coding’. Apelin is downregulated in pulmonary arterial hypertension and heart failure. To replace the missing endogenous peptide, ‘biased’ apelin agonists have been designed that preferentially activate G protein pathways, resulting in reduced β-arrestin recruitment and receptor internalisation, with the additional benefit of attenuating detrimental β-arrestin signalling. Proof-of-concept studies support the clinical potential for apelin receptor biased agonists.

## Highlights

• A biased apelin receptor agonist shows proof-of-concept for utility in the clinic.
• Elabela/Toddler is a new apelin receptor ligand in the human cardiovascular system.
• Enhancing apelin signalling is beneficial in pulmonary arterial hypertension.

## Introduction

Since its discovery in 1993, there is increasing evidence of the importance of the apelin receptor in the mammalian cardiovascular system. This review provides an update of apelin receptor pharmacology, cardiovascular physiology and pathophysiology, and discusses the role of a second receptor ligand, Elabela/Toddler. Finally, there is increasing impetus to develop biased apelin receptor agonists for clinical use.

## Apelin receptor discovery, signalling, and distribution

The apelin receptor (also known as APJ, APLNR, AGTRL1) is a class A GPCR discovered in 1993 based on its sequence similarity with the angiotensin AT_1_ receptor [1]. The apelin receptor did not bind angiotensin II and therefore remained an orphan receptor until the identification of apelin as an endogenous ligand in 1998 [2]. The human (*Homo sapiens*) apelin receptor consists of 380 amino acids and is identified and conserved in many species, including mouse (*Mus musculus*), rat (*Rattus norvegicus*), Western clawed frog (*Xenopus tropicalis*), and zebrafish (*Danio rerio*). To date only one apelin receptor has been shown to exist in mammals, although two subtypes are present in amphibians and fish [3,4], and there are no closely related genes [1].

Apelin responses are in part sensitive to pertussis toxin, consistent with coupling via G_αi_ [5,6] with additional evidence for involvement of G_αq_-linked activation of phospholipase C (PLC) and protein kinase C (PKC) [7]. In cardiac development a role for G_α13_-activated myocyte enhancer factor 2 has been proposed [8]. Interestingly, in the heart the apelin receptor may act as a mechanosensor for stretch in an apelin-independent/G protein-independent manner through recruitment of β-arrestin [9], a protein that initiates receptor internalisation as well as downstream signalling [10]. Indeed, particular residues within the receptor have been identified as being crucial for G protein-independent signalling. Phe 255 and Trp 259 of the rat apelin receptor (corresponding to Phe 257 and Trp 263 of the human receptor) have been reported to be absolutely required for rapid receptor internalisation initiated by the binding of apelin-17 or [Pyr^1^]apelin-13 via an interaction with the C-terminal phenylalanine residue in the peptide ligands [11]. In the C terminus, Ser 348, common across species, is essential for phosphorylation of the receptor following peptide binding, β-arrestin recruitment, and subsequent receptor internalisation [12]. Convincing evidence for apelin receptor heterodimerisation *in vivo* has not yet been obtained in native tissue; however, *in vitro* studies suggest that such receptor complexes may confer novel modes of receptor activation and signalling [13,14].

There is widespread distribution of apelin receptor in peripheral organs including heart, lung, kidney, and placenta [3,6,15–17]. Autoradiographical and immunohistochemical detection of receptor protein localised it to vascular endothelial and smooth muscle cells in addition to cardiomyocytes [18]. Apelin receptor is also present in the central nervous system [16,17] but, because peptides such as apelin are unlikely to cross the blood–brain barrier, the peripheral and central receptors may be considered as separate transmitter systems.

## Apelin receptor ligands

### Endogenous apelin receptor peptides

**Figure 1:**
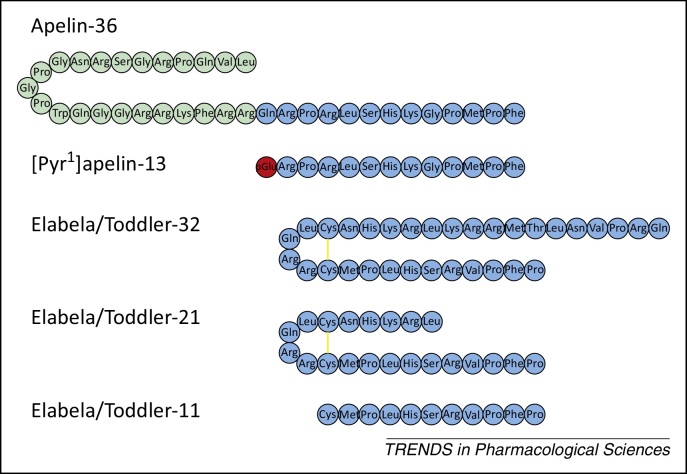
Sequences of apelin and Elabela/Toddler peptides, apelin-36, [Pyr^1^]apelin-13, Elabela/Toddler-32; Elabela/Toddler-21, Elabela/Toddler-11. Three-letter codes of amino acid residues are shown. pGlu, pyroglutamate; yellow lines represent disulfide bridges.

Evidence indicates that apelin acts as a paracrine/autocrine mediator because peptide expression patterns follow that reported for the receptor [16] and, within the human cardiovascular system, the peptide can be detected in cardiac and vascular endothelial cells [19]. The structure of apelin pre-proproteins was deduced following identification of an endogenous 36 amino acid peptide (Figure 1) in bovine stomach extracts [2]. Potential proteolytic cleavage sites predicted that shorter C-terminal apelin peptides, apelin-17, apelin-13 and [Pyr^1^]apelin-13 (Figure 1), may be biologically relevant; these have been synthesised and exhibit varying affinities and potencies for the apelin receptor [4]. However, in human heart [20] and plasma [21], [Pyr^1^]apelin-13 was the predominant isoform detected. Endogenous apelin peptides are derived from the C terminus of the 77 amino acid precursor pre-proapelin, and sequence alignment from human, rat, and mouse indicates that the C-terminal 23 amino acids are fully conserved [4]. Although the synthetic pathways of biologically relevant peptides have not been clarified, it has been shown that direct conversion of the 55 amino acid proapelin to apelin-13 by proprotein convertase subtilisin/kexin 3 (PCSK3 or furin) [22] is possible, and short peptides do not need to be cleaved from intermediate-length peptides such as apelin-36. Following intravenous administration of [Pyr^1^]apelin-13 in rat the C-terminal phenylalanine is cleaved or the peptide is hydrolysed between Pro-10 and Met-11 [23]. Interestingly, apelin-13 and apelin-36 are substrates of angiotensin-converting enzyme (ACE)-2 that removes this C-terminal phenylalanine [24]. This has been assumed to be an inactivating step by generating biologically inert apelin metabolites; however, [Pyr^1^]apelin-13_(1–12)_ retains comparable biological activity to the full-length peptide and its endogenous expression can be localised to endothelial cells [25]. Molecular modelling and biological evaluation of apelin analogues in structure–activity studies reveal that the RPRLS and KGPM sequences together with the RPRL β-turn are necessary for receptor binding [16,26–28], with specific residues within these two sequences being crucial for determining agonist binding [16,29] and potency [16,26,30]. Introduction of unnatural amino acids at the C terminus of the apelin sequence can significantly enhance binding affinity, functional potency, and plasma stability [31].

### Synthetic agonists and biased agonists

Development of synthetic apelin agonists has included cyclisation [32], PEGylation [33], and the synthesis of lipopeptides based on 12-mer sections of the apelin receptor [34]. Although useful as tool compounds, a limitation in translation to clinic is that chronic administration of an agonist will likely cause receptor desensitisation, with subsequent β-arrestin-mediated downregulation and loss of therapeutic efficacy. Therefore, the report of agonists that are biased toward G protein signalling is encouraging. E339-3D6 was described as a peptidomimetic agonist of high molecular weight [35] but has subsequently been shown to be a mixture of polymethylated species, the most abundant of which has been purified and used to develop several modified compounds including G protein biased and non-biased analogues [36]. Removal of the C-terminal phenylalanine of apelin-17 (apelin-17_(1–16)_) or [Pyr^1^]apelin-13 is reported to favour G protein over β-arrestin signalling [25,37]. MM07 is a cyclic apelin peptide that preferentially activates G protein responses with low potency in β-arrestin and receptor internalisation assays. However, of particular significance is that this peptide was an effective dilator in human forearm, with no loss of effect on repeat dosing, and MM07 increased cardiac output in the rat via a load-independent effect, providing proof-of-concept of clinical potential for biased agonists [38]. Intriguingly, whereas the G protein biased MM07, a cyclic 13 amino acid peptide, elicited vasodilatation in human forearm and noradrenaline-constricted hand vein [38], apelin-17_(1–16)_, a linear unmodified peptide, also reported to be G protein biased, lacked vasodilator capacity in an *in vivo* rat renal afferent arteriolar preparation preconstricted with angiotensin II [37]. This may reflect differences in the peptide used and/or the species and vascular bed investigated, or may indicate a particular role for apelin receptor-mediated β-arrestin signalling in the reversal of angiotensin II constriction. Small-molecule agonists are also in development, and the first non-peptide agonist ML233 [39] was reported to weakly inhibit forskolin-stimulated cAMP production and exhibited full agonist activity in β-arrestin recruitment in cells expressing the apelin receptor.

### Apelin receptor antagonists

MM54, designed using a bivalent ligand approach, was the first competitive apelin receptor antagonist reported with nanomolar affinity for cloned human apelin receptor but with no affinity for the AT_1_ receptor [40]. ML221 is a small-molecule antagonist discovered from a high-throughput screening programme with micromolar potency; however, its utility is limited by its poor solubility and rapid metabolism [41]. A small-molecule antagonist that blocks the CXC motif chemokine receptor CXCR4, ALX40-4C, also blocks the apelin receptor as a coreceptor for HIV [42].

## Physiological functions of apelin receptor signalling

A diverse range of physiological functions of apelin signalling have emerged since its discovery. This review focuses on the regulation of the cardiovascular system by apelin and its receptor. For reviews of other important functions of apelin peptides including fluid homeostasis and metabolism see [4,43–45], and for the role of the apelin receptor as a coreceptor for HIV infection see [46].

### Apelin and apelin receptor knockout mice

Important insights into the cardiovascular roles of apelin receptor signalling have been obtained from the generation of apelin [47–49] and apelin receptor [49–51] knockout mice. Apelin knockout mice have normal heart morphology and blood pressure [47,49] but show a modest reduction in basal cardiac contractile function [49]. These animals develop impaired cardiac contractility with age [47] and have a marked decrease in exercise capacity [49]. They develop severe heart failure (HF) under pressure overload, that can be rescued by inhibition of the AT_1_ receptor or infusion of angiotensin 1–7 [47,52], and exhibit worsened pulmonary hypertension under hypoxia [53]. In apelin knockouts, following acute myocardial infarction infarct size and mortality were increased [54], and with carotid ligation neointimal lesion area was reduced [55].

Homozygous apelin receptor knockout mice are born in sub-Mendelian ratio [49–51] owing to cardiovascular developmental defects, including poorly looped hearts with aberrantly formed right ventricles, defective atrioventricular cushion formation, and deformed vasculature of the yolk sac and the embryo [8]. The surviving adult mice show normal systolic blood pressure [50] and modest reduction in basal cardiac contractile function [49]. There was a striking decrease in exercise capacity [49], and these animals are more sensitive to angiotensin II following ACE inhibition [50]. Interestingly, apelin receptor knockout mice showed reduced cardiac hypertrophy and HF in response to pressure overload [9]; however, this is an apelin peptide-independent effect (see sections below on physiological function and HF). Evidence that apelin receptor activation opposes the angiotensin system is supported by the apelin receptor/AT_1_ receptor double-knockout mouse that exhibits elevated baseline blood pressure [50]. Overall, information from deletion of peptide or receptor would indicate that the apelin system is important for normal cardiovascular development and function but is crucial for appropriate protective responses under conditions of cardiovascular stress. However, it is clear there are unexplained discrepancies in the phenotypes of the peptide and receptor knockouts (Table 1).

### Discovery of Elabela/Toddler

A possible explanation for these phenotypic discrepancies, particularly in cardiovascular development, may come from the recently discovered Elabela/Toddler, whose gene, *apela*, was identified in a conserved but previously designated non-coding region of the genome [56–58]. *Apela* mutant zebrafish exhibit severe developmental defects including rudimentary or absent heart formation, reminiscent of the *apelin receptor* phenotypes. In addition, the Elabela/Toddler and apelin receptor spatiotemporal expression pattern, mutant rescue, and receptor internalisation experiments also suggest that Elabela/Toddler may be a ligand of the apelin receptor. These studies predicted the existence of three mature Elabela/Toddler peptides with 32, 21, and 11 amino acids (Figure 1) based on peptidase cleavage [56,57].

A role for Elabela/Toddler beyond development can be inferred from expression in adult human heart and blood vessels, where Elabela/Toddler is localised to the endothelium and nanomolar affinity of Elabela/Toddler was demonstrated for the human apelin receptor [59]. In addition, mRNA for Elabela/Toddler is expressed in human stem cells, prostate, and kidney [56,60]. Elabela/Toddler activated signal transduction pathways downstream of overexpressed human apelin receptor, promoted angiogenesis, and induced vasodilatation in mouse aorta [60]. More importantly, Elabela/Toddler attenuated the angiotensin II-induced pressor effect in mouse *in vivo* [59].

### Apelin receptor signalling in the cardiovascular system

**Figure 2:**
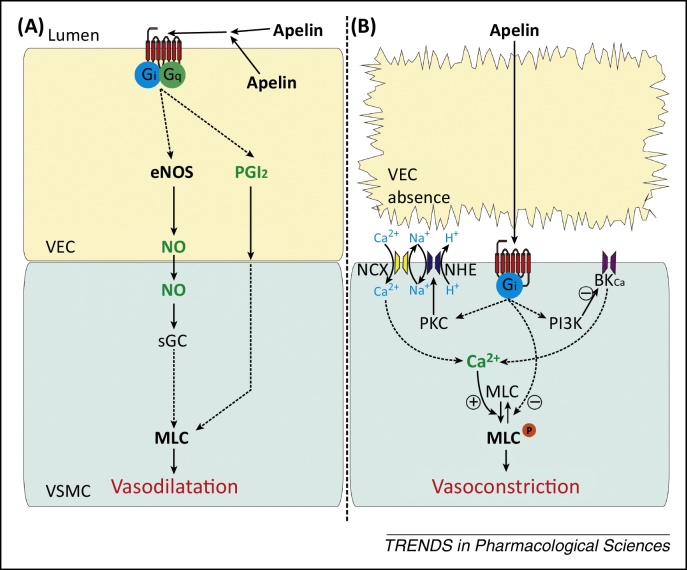
Proposed mechanisms of vasodilator and vasoconstrictor effects of apelin. **(A)** Apelin activates endothelial apelin receptors producing vasodilatation by pathways including release of NO. **(B)** In the absence of endothelium, apelin activates apelin receptors on the underlying smooth muscle to cause vasoconstriction. The apelin receptor is represented by the cell surface seven-transmembrane receptor in red; broken arrows indicate unspecified intermediate steps; + and − signs indicate positive and negative regulation, respectively. Abbreviations: BK_Ca_, large-conductance Ca^2+^-activated K^+^ channel; eNOS, endothelial nitric oxide synthase; MLC, myosin light chain; MLC^P^, phosphorylated myosin light chain; NCX, Na^+^/Ca^2+^ exchanger; NHE, Na^+^/H^+^ exchanger; NO, nitric oxide; PGI_2_, prostacyclin; PI3K, phosphatidylinositol 3-kinase; PKC, protein kinase C; sGC, soluble guanylyl cyclase; VEC, vascular endothelial cells; VSMC, vascular smooth cells.

*In vivo*, infusion of apelin into human forearm elicited vasodilatation [38,61] with some evidence for contribution from nitric oxide (NO) release [61]. Systemic apelin reduced blood pressure in anaesthetised and conscious rats [38,62–64], in wild type but not apelin receptor-deficient mice [50], and reduced mean arterial pressure and peripheral vascular resistance in humans [65]. In human isolated blood vessels apelin has also been shown to produce both NO-dependent [66] and NO-independent prostanoid-dependent responses [20], and stimulated NO synthase (NOS) activity and expression in rat aorta [67] (Figure 2A). Removal of the endothelium results in vascular contraction *in vitro* [3,20]. These opposing endothelium-dependent and -independent effects may reflect the activation of apelin receptors on endothelial versus smooth muscle cells. Vasoconstriction may involve phosphorylation of myosin light chain (MLC) via protein kinase C (PKC), Na^+^/H^+^ exchanger (NHE), and Na^+^/Ca^2+^ exchanger (NCX), together with inhibition of MLC phosphatase [68] or inhibition of large-conductance Ca^2+^-activated K^+^ channels via phosphatidylinositol 3-kinase (PI3K) [69] (Figure 2B). Whether apelin-mediated vasoconstriction is physiologically relevant is debatable, but it may be of relevance in conditions of endothelial dysfunction. In addition to its peripheral actions, apelin may also modulate blood pressure when administrated centrally, particularly when injected into the subfornical organ, which detects circulating signals [70]. Furthermore, apelin and its receptor may interact with other regulators of vascular tone. For example, apelin counteracts the vasoconstrictor action of angiotensin II by a NO-dependent mechanism [71], as supported by the apelin receptor knockout mouse [50].

In addition to an effect on the vasculature, apelin may be an important regulator of cardiac contractility via activation of the receptor present on cardiomyocytes [18]. A positive inotropic action of apelin has been demonstrated *in vivo* in rats [72–74], mice [75], and humans [65] and is supported by the decreased basal contractility observed in apelin and apelin receptor knockout mice [49]. In addition, chronic apelin infusion resulted in increased cardiac output without hypertrophy in mice [75]. In human [20] and rat [76] cardiac tissue apelin is reported to be a more-potent positive inotrope than for example endothelin-1, adrenomedullin, or urotensin-II.

**Figure 3:**
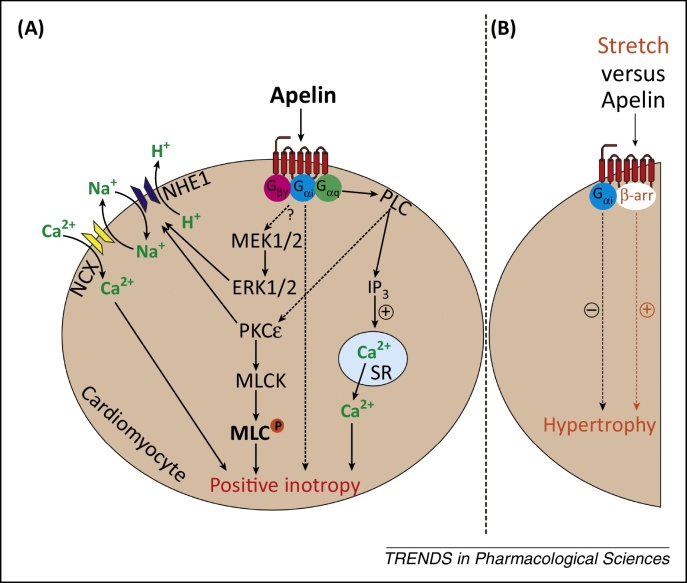
Proposed mechanisms of apelin-induced positive inotropy and apelin-independent stretch-induced hypertrophy. **(A)** Apelin activates apelin receptors on cardiomyocytes to induce positive inotropy. **(B)** The apelin receptor mediates β-arrestin-dependent signalling (orange) to cause hypertrophy in response to stretch, whereas apelin-activated G_αi_ signalling (black) is protective. The apelin receptor is represented by the cell surface seven-transmembrane receptor in red; broken arrows indicate unspecified intermediate steps; broken arrow with? mark indicates unknown intermediate steps; + and − signs indicate positive and negative regulation, respectively. Abbreviations: β-arr, β-arrestin; ERK1/2, extracellular signal-regulated kinase 1/2; IP_3_, inositol trisphosphate; MEK1/2, mitogen-activated protein kinase kinases 1/2; MLC, myosin light chain; MLCK, myosin light chain kinase; NCX, Na^+^/Ca^2+^ exchanger; NHE1, Na^+^/H^+^ exchanger 1; PKCɛ, protein kinase Cɛ; PLC, phospholipase C; SR, sarcoplasmic reticulum.

In the heart, apelin signals via phospholipase C, PKCɛ, NHE, NCX [76], and extracellular signal-regulated kinase 1/2 (ERK1/2), leading to activation of MLC kinase, increased myofilament Ca^2+^ sensitivity [77], and increased sarcomere shortening [78]. In addition, apelin may induce positive inotropy by increasing [Ca^2+^]_i_ transients and Ca^2+^ availability independently of PKC [79,80] (Figure 3A).

### The apelin receptor and cardiovascular development

The apelin receptor appears to be essential for normal cardiac development, as shown by the severe cardiovascular phenotype of the receptor knockout mice [8]. A mutation that causes a tryptophan to leucine substitution in the second transmembrane domain of zebrafish apelin receptor results in a deficit in cardiomyocyte number and may manifest in pericardial oedema and absence of a heart [81]. It is now known that Elabela/Toddler is the crucial developmental regulator in zebrafish; a murine *apela* knockout is anticipated and will confirm whether this is also the case for mammals. However, appropriate expression of apelin may also be required for cardiac progenitor cells [81–83]. Moreover, normal vascular development also requires apelin functioning as an angiogenic factor [84,85].

## Targeting the apelin receptor in disease

Much of the current interest in apelin and the cardiovascular system has focussed on the potential therapeutic benefit of targeting the apelin receptor in pulmonary arterial hypertension (PAH) and HF. By contrast, there is currently less evidence supporting the contribution of the apelin pathway in other cardiovascular conditions (but see reviews [4,86]).

### Pulmonary arterial hypertension

**Figure 4:**
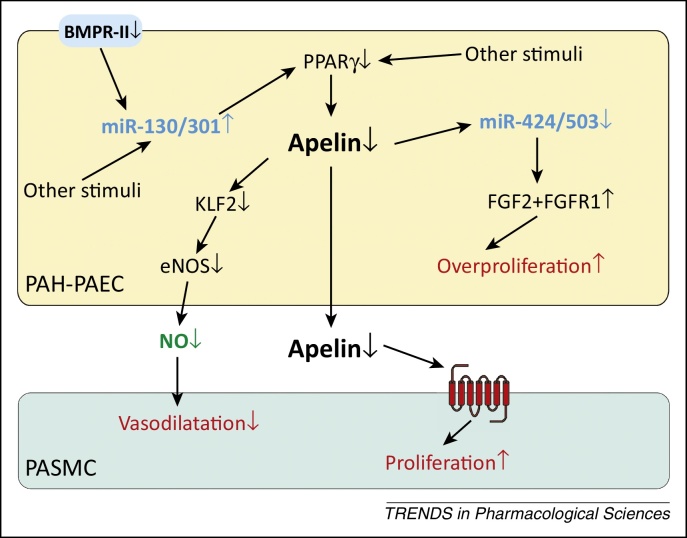
Summary of reported causes and consequences of reduced vascular apelin levels in pulmonary arterial hypertension (PAH) lung. In PAH, reduced BMPR-II expression and function, together with other stimuli, cause a reduction in endothelial PPARγ and, as a consequence, a reduction in the expression of the PPARγ transcriptional target apelin. Loss of apelin results in disinhibition of FGF2-stimulated endothelial proliferation and smooth muscle cell proliferation, with a potential reduction in apelin-mediated vascular dilatation. miRNA families may act as important pathway components. Increase and decrease of the pathway components in PAH are denoted by upward and downward arrows. The apelin receptor is represented by the cell surface seven-transmembrane receptor in red. Abbreviations: BMPR-II, bone morphogenic protein receptor type II; eNOS, endothelial nitric oxide synthase; FGF2, fibroblast growth factor 2; KLF2, kruppel-like factor 2; NO, nitric oxide; PAH-PAEC, pulmonary arterial endothelial cells in pulmonary arterial hypertension; PASMC, pulmonary arterial smooth muscle cells; PPARγ, peroxisome proliferator-activated receptor γ.

PAH is a fatal disease characterised by complex remodelling of the pulmonary vasculature, that results in increased pulmonary arterial pressure and vascular resistance, leading to right ventricular hypertrophy and eventually death from right ventricular failure [87]. Circulating levels of apelin [53,88] and apelin expression in pulmonary arterial endothelial cells (PAECs) [89] and pulmonary microvascular endothelial cells (PMVECs) [90] are reduced in human PAH. In agreement, apelin deficiency exacerbates pulmonary hypertension in response to hypoxia in mice [53]. Patients with PAH show decreased expression and function of bone morphogenic protein receptor type II (BMPR-II) with or without associated BMPR-II mutations [87]. Loss of BMPR-II function is associated with upregulation of the miRNA miR-130/301 family [91] and reduction in the transcriptional activity of PPARγ, whose targets include apelin [90] (Figure 4). Importantly, despite the reduction of apelin expression in PAH, the receptor is present and can be targeted therapeutically [89,92]. The potential benefit of apelin agonists is demonstrated by the ability of exogenous [Pyr^1^]apelin-13 to prevent the development of monocrotaline-induced PAH [93]. Furthermore, apelin reverses established mild PAH pathologies in mice with endothelial PPARγ deletion [90]. In addition, several studies using agents that indirectly augment the apelin signalling pathway are beneficial in PAH. For example, inhibiting the miR-130/301 family resulted in the reversal of established PAH [91], as did administration of tacrolimus (FK506) or the endogenous elastase inhibitor elafin, which reportedly increased apelin expression through enhancing BMPR-II signalling [94,95]. Administration of miR424 and miR503, which are anti-proliferative miRNAs regulated by apelin (Figure 4), can also prevent and rescue PAH [89].

Apelin may be beneficial in PAH by influencing multiple aspects of the disease. First, enhancing apelin signalling lowered indices of pulmonary arterial pressure in animal models [90,91,93]. This may be a consequence of apelin-induced enhancement of pulmonary NOS expression [53] and/or an attenuation of expression of vasoconstrictors such as endothelin-1 and angiotensin II [93]. These effects may rectify the imbalance between vasoconstrictors and vasodilators in PAH.

Second, apelin may resolve the imbalance between proliferation and apoptosis in PAH, thus limiting the extent of vascular remodelling. Apelin promotes proliferation in normal PAECs [89,90]; however, proliferation is attenuated in PAECs from PAH [89], and this may be a consequence of normalised expression of miR-424 and miR-503 and their targets fibroblast growth factor 2 and its receptor [89] (Figure 4). However, apelin promotes survival in both normal and PAH PMVECs [90], suggesting that further investigation will be necessary to clarify whether the role of apelin is to restore normal endothelial function and/or to suppress unregulated endothelial cell proliferation in PAH. A recent study reported that apelin potentiated the ATPase activity of the endothelial enzyme CD39 *in vitro* and *in vivo*, whereas suppression of CD39 gave rise to apoptosis-resistant PAECs which may be responsible for advanced vascular lesions in PAH [96]. By contrast, the effect of apelin on pulmonary arterial smooth muscle cells is consistently anti-proliferative and pro-apoptotic [89,90] and anti-migratory [96], indicating that loss of apelin in PAH results in reduced suppression of smooth muscle cell proliferation that is a hallmark of the disease. Overall, apelin protects against arterial muscularisation and the loss of microvessels *in vivo* [53,89–91].

Finally, in addition to effects on the pulmonary vasculature, apelin may also beneficially augment right ventricular performance in PAH. Apelin increased contractility in trabeculae from failing right ventricle caused by hypoxia-induced pulmonary hypertension [79], and did not induce cardiac or cardiomyocyte hypertrophy [75]. More importantly, *in vivo* studies using PAH models consistently reported reduced right ventricular hypertrophy in response to administered apelin [90,93].

### Left ventricular HF

In addition to right ventricular failure in PAH, enhancing apelin receptor signalling may also be beneficial in left ventricular HF. In humans, plasma apelin levels are unaltered or increased in early stages of HF [97,98], but reduced in advanced disease [97,99–101]. These results suggest an initial compensatory increase in apelin to improve cardiac contractility but, as the disease progresses, apelin is reduced or lost. Interestingly, apelin peptide levels are elevated in plasma after cardiac resynchronisation therapy [101] and in cardiac tissue following ventricular offloading [97]. In animal models of HF, the initial preservation and later downregulation of apelin in severe disease are also observed, recapitulating the human condition [73,102–104]. More importantly, apelin knockout mice develop progressive left ventricular dysfunction with age and severe HF with pressure overload [47].

The effect of HF on apelin receptor expression is less clear, and levels were reportedly unchanged or reduced in patients with cardiomyopathies [99], and unaltered, reduced, or increased in animal models of HF [73,74,102–104]. Of interest, the apelin receptor was the most upregulated of ∼12 000 genes following ventricular offloading [97]. Several apelin receptor polymorphisms have been reported; however, only the 212A allele of a 5′ untranslated region polymorphism has been associated with slowing of HF progression [105]. Furthermore, an apelin-independent stretch-sensitive function of the receptor may mediate myocardial hypertrophy and HF in response to chronic pressure overload via β-arrestin signalling, whereas apelin-induced G_αi_ signalling is protective [9], indicating a potential advantage of G_αi_-biased agonists in this condition [38] (Figure 3B).

Therefore, as predicted, exogenous apelin is beneficial in a range of animal HF models [72–74,103,104,106], and retained or enhanced the inotropic action of apelin [72,78,79] and attenuated left ventricular hypertrophy [107]. Similarly, in HF patients, infusion of apelin locally caused vasodilatation [65,108] despite evidence of endothelial dysfunction indicated by attenuated dilatation induced by acetylcholine [108]. Systemically, apelin increased cardiac index, reduced peripheral vascular resistance, and moderately lowered mean arterial pressure, without an increase in heart rate [65]. These actions of apelin were preserved during prolonged infusion and under conditions of renin/angiotensin system activation [108].

Emerging evidence suggests that apelin receptor signalling is beneficial beyond established HF and confers cardioprotection during myocardial injury. Cardiac apelin expression was upregulated in response to hypoxia/ischaemia [109,110]. Apelin deficiency worsened outcome in myocardial infarction and ischaemia–reperfusion injury [54]. By contrast, apelin administration during reperfusion or post-infarct reduced infarct size and ameliorated heart dysfunction via cardioprotective mechanisms, including activation of the reperfusion injury salvage kinase pathway, which may involve PI3K-protein kinase B or ERK, reduction of apoptosis and reactive oxygen species production, and increased NO production [111–114].

## Concluding remarks

The discovery of a second peptide, Elabela/Toddler, provides an elegant explanation of why apelin peptide knockout does not recapitulate the dramatic failure of the cardiovascular system to develop following deletion of the apelin receptor gene. The identification of *apela* within a region of the genome hitherto classified as ‘non-coding’ may hold the key to the identification of endogenous ligands for the remaining 130 orphan GPCRs [115]. It is remarkable that two peptides with so little sequence similarity can activate a single receptor, and the challenge will be to understand how these act spatiotemporally. Compelling evidence suggests that the apelin receptor is one of the first GPCR pathways tractable to the design of biased agonists with proof-of-concept for utility in the clinic. Further studies will reveal whether such biased agonists will revolutionise our understanding of pharmacology and drug discovery in the future.

## Figures and Tables

**Table 1:** Comparison of apelin receptor-related phenotypes

*Apln* knockout mice	*Aplnr* knockout mice	*Apela* mutant zebrafish
Mendelian birth ratio	Loss of homozygous mice	Loss of mutants
Normal heart morphology	Severe cardiac developmental defects	Rudimentary or no heart
Normal blood pressure	Normal blood pressure	
Modest decrease in basal cardiac contractility	Modest decrease in basal cardiac contractility	
Marked decrease in exercise capacity	Marked decrease in exercise capacity	
Severe heart failure in response to pressure overload	Markedly reduced heart failure in response to pressure overload	
